# Revolutionizing Class II Division 1 Malocclusion Treatment With Forsus Appliance: A Clinical Case

**DOI:** 10.7759/cureus.66930

**Published:** 2024-08-15

**Authors:** Srushti Atole, Ranjit Kamble, Vikrant V Jadhav, Japneet Kaiser, Shefali Singh, Samiksha Tidke

**Affiliations:** 1 Department of Orthodontics and Dentofacial Orthopedics, Sharad Pawar Dental College and Hospital, Datta Meghe Institute of Higher Education and Research, Wardha, IND

**Keywords:** class ii malocclusion, fixed functional appliance, mandibular advancement, orthodontics, forsus appliance

## Abstract

Angle's Class II Division 1 malocclusion is illustrated as a prominent maxilla along with protrusive maxillary anteriors, mandibular retrognathism, or both, often leading to functional and aesthetic concerns. Effective management of this condition in growing patients typically involves a combination of functional and orthodontic appliances to correct dental and skeletal discrepancies. Treating this malocclusion in the deceleration stages of growth is often challenging for orthodontists. This case report exemplifies the potency of Forsus appliance in addressing Class II Division 1 malocclusion in growing patients, underscoring its role in achieving favorable orthodontic outcomes.

## Introduction

Class II malocclusion is commonly referred to as mandibular distocclusion [1]. Approximately one-third of people suffer from Class II malocclusion which is usually a common condition in orthodontics [2]. The malocclusion represented a mandibular retruded position with a maxillary jaw base along with other skeletal and dental abnormalities which can negatively impact both the functional status and appearance of the face [3]. Angle Class II malocclusion is further differentiated into Divisions 1 and 2 based on the correlation of the anterior segment. There may be mandibular retrusion, maxillary protrusion, and both jaws in malocclusions with Class II Division 1, as well as abnormal dental relationships and facial esthetic disorders [4].

Class II malocclusions are comparatively complicated malocclusions to treat in the orthodontic profession. Various functional appliances exist to treat such conditions to improve the esthetics and function of malocclusion with Skeletal Class II conditions [5,6]. A variety of "myofunctional appliances," for example, the activator twin block, and Frankel's regulator can correct Skeletal Class II malocclusions during active growth [7]. There is disagreement among various schools of thought regarding the management of deceleration stage Class II conditions [8]. Certain fixed functional appliances turned out to be effective in managing certain skeletal defects even during the deceleration of the growth phase [9].

A wide spectrum of options is available for managing Class II malocclusions. Patients with Angle's Class II condition can be treated using fixed or removable functional appliances based on their sagittal discrepancy, cooperation, and status of growth. Individuals with Class II conditions due to retrusion of the mandible and have finished growth are generally corrected by the use of fixed functional appliances that need minimal patient cooperation [10,11]. Many Class II correctors that minimize or do entirely with the need for patient compliance have recently been introduced. The Forsus fixed appliance therapy was chosen for this patient because she presented with complete growth. Appliance encouraged significant changes in the dentoalveolar structure as well as subtle but noticeable alterations in the skeletal structure. This functional appliances also shows appreciable improvement in external soft tissues including improvement in facial convexity, increase in nasolabial angle, mentolabial angle, and Z-angle [12]. 

## Case presentation

**Figure 1:**
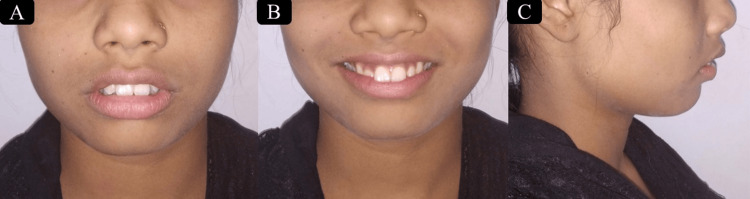
Pretreatment extraoral photographs. (A) Frontal-at rest position. (B) Frontal-at smiling. (C) Profile-at rest

A young female patient of age 16 years visited to the Orthodontics and Dentofacial Orthopedics Department with the main concern of forwardly placed anterior teeth. While examining extraoral features, it is noticed that the patient has a mesoprosopic face form and a grossly symmetrical face with potentially competent lips. She had a convex profile, deep mentolabial sulcus (MLS), and normal nasolabial angle) (Figure 1).

**Figure 2:**
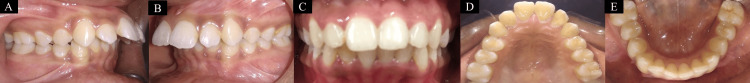
Pretreatment intraoral records. (A) Right buccal. (B) Left buccal. (C) Front occlusion. (D) Maxillary occlusal. (E) Mandibular occlusal

The examination of intraoral structures revealed whole permanent dentition were found in the upper and lower arch apart from the third molars and on canine and molar relation observed on both sides. Moderate proclination was observed in the maxillary arch (Figure 2).

**Figure 3:**
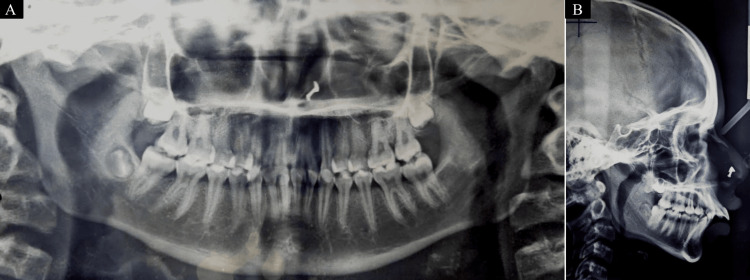
Pretreatment records (radiographs). (A) Orthopantomogram. (B) Lateral cephalogram

The patient's orthopantomogram revealed the existence of third molars except in the third quadrant with no significant abnormality. A cephalometric examination revealed the patient was in cervical vertebral maturation index (CVMI) stage V (maturation) accompanied by Skeletal Class II bases because of a retrusive mandible. The patient had a horizontal growth pattern. The lateral cephalogram depicts proclined upper and lower anteriors (Figure 3) (Table 1). 

**Table 1:** Pretreatment cephalometric readings S: Sella point; N: nasion; A: A point; B: B point; WITS: Wits appraisal; U1: upper central incisor; L1: lower central incisor; FMA: Frankfort mandibular plane angle; IMPA: incisor mandibular plane angle; FMIA: Frankfort mandibular incisor plane angle

Parameters	Pretreatment	Reference
SNA	83^◦^	Orthognathic maxilla
SNB	77^◦^	Retrognathic mandible
ANB	6^◦^	Skeletal Class II
WITS	6 mm	Skeletal Class II
U1 TO NA (degrees)	32^◦^	Proclined UI
U1 TO NA (mm)	7 mm	Protruded UI
L1 TO NB (degrees)	29^◦^	Proclined LI
L1 TO NB (mm)	5 mm	Protruded LI
FMA	24^◦^	Horizontal growth pattern
IMPA	96^◦^	Proclined LI
FMIA	60^◦^	Proclined LI
Maxillary length	74 mm	Reduced
Mandibular length	92 mm	Reduced

Diagnosis

Based on clinical and radiographic examinations, the patient was diagnosed as Skeletal Class II malocclusion with Angle's Class II Division 1 with a horizontal growth pattern. The following table depicts the treatment objectives of the presented case (Table 2).

**Table 2:** Treatment objectives

	Sagittal	Vertical
Skeletal	Correction of retrognathic mandible	
Dental	1. Correction of proclination in maxillary and mandibular anteriors	1. Correction of deep curve of spee
	2. Correction of overjet	2. Correction of overbite
	3. To bring off a Class I canine and molar relationship bilaterally	
Soft tissue	To bring off straight profile	
	Correction of deep mentolabial sulcus	
	To get competent lips	

Treatment plan

Phase I includes patient motivation and education.

Oral Prophylaxis

Phase II includes fixed mechanotherapy using MBT 0.022 × 0.028 slot.

Non-extraction Treatment Plan

To correct mandibular retrusion and to attain Class I canine and molar relation, Forsus functional appliance was opted that helped in mandibular growth modulation. Phase III includes finishing and detailing. For retention, Hawley’s retainer was used. 

Treatment progress

**Figure 4:**
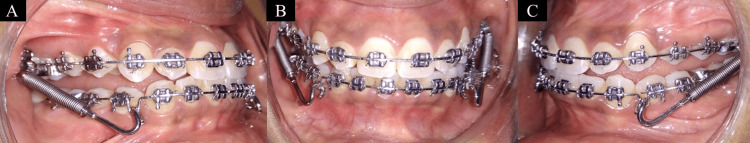
Intraoral records after placement of Forsus fixed appliance. (A) Right occlusion. (B) Front occlusion. (C) Left occlusion

Treatment was started with bonding in both arches using 0.022 × 0.028 slot McLaughlin, Bennett, and Trevisi (MBT) brackets. Initial levelling and alignment were done using 0.016 NiTi arch wire to 0.017" × 0.025" NiTi wire. After complete alignment, fixed functional therapy started. Fixed functional appliance Forsus was given to correct the position of the mandible and to bring off Class I canine and molar relationship (Figure 4).

After attaining the Class I canine and molar relationship, the settling phase was started. Currently, occlusion on both the right and left side is completely settled (Figure s 5-7).

**Figure 5:**
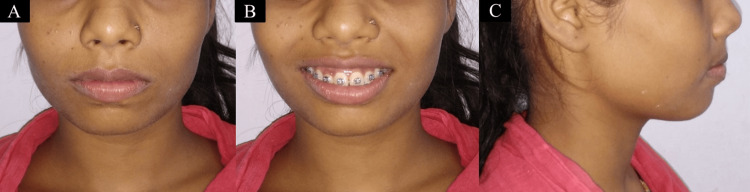
Current status of the patient. (A) Front nonsmiling. (B) Front smiling. (C) Profile

**Figure 6:**
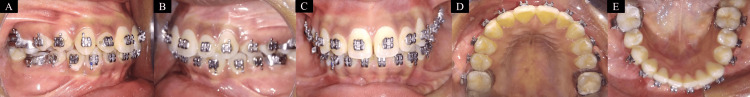
Current intraoral status. (A) Right occlusion. (B) Left occlusion. (C) Front occlusal. (D) Maxillary occlusal. (E) Mandibular occlusal

**Figure 7:**
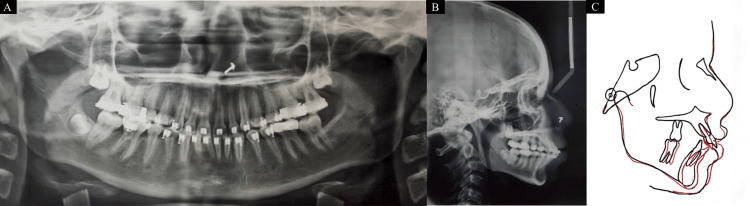
Current (A) orthopantomogram. (B) Lateral cephalogram. (C) Pre- and posttreatment superimpositions

At present, finishing and detailing are ongoing for both jaws. After debonding, a permanent lingual retainer along with Hawley's retainer will be given.

Treatment results

Every planned objective of treatment was successfully achieved at the end of 15 months of active treatment. Skeletally, a normal mandible position was achieved. Correction of increased inclination of the anteriors was obtained along with normal overjet and overbite. Also, planned canine and molar relations were achieved. The patient profile was also improved. The pretreatment and posttreatment cephalometric values also confirm the improvement (Table 3). At present, the case is in the finishing phase.

**Table 3:** Pre- and posttreatment cephalometric analysis S: Sella point; N: nasion; A: A point; B: B point; WITS: Wits appraisal; U1: upper central incisor; L1: lower central incisor; FMA: Frankfort mandibular plane angle; IMPA: incisor mandibular plane angle; FMIA: Frankfort mandibular incisor plane angle

Parameters	Pretreatment	Posttreatment
SNA	83^◦^	82^◦^
SNB	77^◦^	80^◦^
ANB	6^◦^	2^◦^
WITS	6 mm	2 mm
U1 TO NA (Degrees)	32^◦^	28^◦^
U1 TO NA (mm)	7 mm	3 mm
L1 TO NB (Degrees)	29^◦^	25^◦^
L1 TO NB (mm)	5 mm	3 mm
FMA	24^◦^	25^◦^
IMPA	96^◦^	95^◦^
FMIA	60^◦^	60^◦^
Maxillary length	74 mm	74 mm
Mandibular length	92 mm	98 mm

## Discussion

Treating Skeletal Class II malocclusion has always been challenging for orthodontists, particularly during the deceleration stages of growth [13]. A removable functional appliance can be very effective, but predictability requires the patient's cooperation for it to work. Moreover, these appliances present several difficulties when it comes to other functions, such as speech. This problem has been addressed by the development of fixed bite-jumping appliances [4]. Orthodontists have utilized a lot of fixed functional appliances over time, but few have demonstrated satisfactory patient acceptance and outcomes [14,15].

The "Forsus Fatigue Resistant Device" (3M Unitek, Monrovia), introduced by William Vogt in 2001, consists of three separate pieces designed to manage Class II conditions. It corrects Class II malocclusion very well by modifying the skeletal and dentoalveolar structures in harmony [16]. The appliance is made up of a rod and spring module. The push rods are connected to the mandibular archwire distal to the canine bracket, while the spring module is fixed to the buccal tube of the upper first molar. Depending on the desired length, the appliance is purchasable in four different dimensions: 28 mm, 31 mm, 34 mm, and 37 mm. The appliance's size needs to be chosen so that once installed, the mandible closes in a Skeletal Class I posture [16]. 

According to a study by Jones [17], the Forsus fatigue-resistant device (FFRD) causes mandibular molars in the group of Forsus to move considerably mesially, causing overall correction of molar and overall molar correction in the Forsus group in comparison to Class II elastics. Additionally, the overall results of fixed orthodontic therapy against FFRD in the correction of Class II malocclusion were evaluated by Franchi and Bacetti [10]. They concluded that FFRD demonstrated notable modifications in the maxillomandibular relations, which had a maxilla-restraining effect.

The patient presented in this case was with Class II malocclusion due to retrusion of the mandible and increased overjet. After considering the patient's age and positive VTO, a nonextraction treatment plan with a fixed functional appliance was opted for. Choosing growth modulation as the modality of treatment in this instance had the benefit of avoiding the need for premolar extraction. The Forsus appliance is taken into consideration for the correction of Class II malocclusion because of its advantages over the other appliances. The use of Forsus helps to minimize the treatment duration of the fixed appliance as well as functional therapy, collaborated in the single phase of treatment. There won't be any constraints because of patient cooperation. The appliance results in a beneficial conversion in the soft tissue profile because of correction in face convexity, even though dentoalveolar effects play a vital role in bringing about Class II correction.

## Conclusions

Even with the current debates about functional appliances and growth modulation, functional appliances can still be very useful tools for treating Class II malocclusions. Functional appliances play a crucial role in managing Class II malocclusion through growth modulation. Growth modulation reduces the need for permanent tooth extractions and potentially orthognathic surgery later in life. The Forsus fixed functional appliance is an effective and efficient tool in the management of Class II Division 1 malocclusion. Its ability to promote mandibular growth and improve dental and skeletal relationships makes it a valuable option in contemporary orthodontic practice.
